# Intratumoral reciprocal expression of monocarboxylate transporter 4 and glypican-3 in hepatocellular carcinomas

**DOI:** 10.1186/s13104-019-4778-y

**Published:** 2019-11-09

**Authors:** Kenji Yorita, Akinobu Ohno, Takahiro Nishida, Kazuhiro Kondo, Toshihiko Ohtomo, Hiroaki Kataoka

**Affiliations:** 1 Section of Oncopathology and Regenerative Biology, Department of Pathology, Faculty of Medicine, University of Miyazaki, 5200 Kihara, Kiyotake, Miyazaki, 889-1692 Japan; 2 Community Medical Center, University of Miyazaki Hospital, Miyazaki, Japan; 3 Project Management Department, Chugai Pharmaceutical Co., Tokyo, Japan

**Keywords:** Glypican-3, Hepatocellular carcinoma, Monocarboxylic acid transporter, MCT4

## Abstract

**Objective:**

We previously reported the identification of monocarboxylate transporter 4 (MCT4) and glypican-3 (GPC3) as prognostic factors for hepatocellular carcinoma (HCC), which are now considered significant poor prognostic factors for the disease. This study aimed to clarify the detailed interaction of these two factors in HCC to improve our understanding of aggressive HCC phenotypes. A total of 225 Japanese patients with HCC from our previous study were subjected to immunohistochemical analyses.

**Results:**

The number of MCT4-positive (MCT4+) HCC cases was 47 (21%), and most MCT4+ HCC showed high GPC3 expression (94%, 44/47 cases). In 44 MCT4+/GPC3+ HCC cases, intratumoral heterogeneity of GPC3 or MCT4 expression was further evaluated. We observed reciprocal (inverse), synergistic, mixed reciprocal and synergistic, or irrelevant interaction of MCT4 and GPC3 expression in 29 (66%), 5 (11%), 1 (2%), and 9 cases (21%), respectively. The cases exhibiting reciprocal expression of both markers tended to have cirrhosis without a history of neoadjuvant therapy. In summary, although MCT4+ HCC cases are mostly GPC3+, intratumoral expression patterns of MCT4 and GPC3 are frequently reciprocal each other, suggesting that dual targeting of MCT4 and GPC3 may achieve a better antitumor effect for MCT4+ HCC cases.

## Introduction

Liver cancer is the leading cause of cancer death worldwide and is the second leading cause of cancer death in men [1]. As hepatocellular carcinoma (HCC) is the most common liver malignancy, the molecular mechanism of its malignant phenotype has been a focus of intensive investigation. We previously reported the identification of prognostic factors for HCC, including glypican-3 (GPC3) [2, 3] and monocarboxylate transporter 4 (MCT4) [4]. GPC3 is an oncofetal glycoprotein connected to the cell membrane via a glycosylphosphatidylinositol anchor [5] and regulates some signaling activities including canonical Wnt signaling [6]. GPC3 is highly expressed in HCC and considered to play a role in cancer invasion and progression; accordingly, GPC3 is also a promising diagnostic and therapeutic marker for HCC. Recent meta-analyses have reported that GPC3 expression is significantly associated with poor prognosis in patients with HCC [7, 8]. Furthermore, we previously showed that circumferential membranous expression of GPC3 might indicate a poor outcome in patients with HCC [2]. On the other hand, MCT4, which is an emerging prognostic marker for various cancers [9], facilitates transmembrane transport of short-chain fatty acids, such as pyruvate and lactate, to prevent intracellular acidosis associated with increased glycolysis [10]. Enhanced MCT4 expression may represent an adaptation to a hypoxic HCC microenvironment [11, 12]. In addition, MCT4 was reported to be colocalized with CD147 [13], and we previously reported synergistic interaction of MCT4 and CD147 in HCCs [4]. As CD147 induces expression of matrix metalloproteases [14, 15], MCT4-positive (MCT+) HCC is postulated to show more aggressive behavior in association with CD147. In fact, we first reported that patients with HCC expressing MCT4 had significantly worse prognosis [4]. This trend of MCT4 in HCCs has been confirmed by other researchers [16, 17].

We previously reported that MCT4+ HCC cases were mostly GPC3 positive [4], and in the double-positive cases, MCT4+ HCC cells may show circumferential membranous GPC3 immunoreactivity [4]; however, this trend of synergistic immunoreactivity in the double-positive HCC was less pronounced in subsequent detailed examination using serial sections of each case. Herein, to improve our understanding of aggressive phenotypes of HCC, this study aimed to clarify the intratumoral heterogeneity and interaction of these two prognostic factors in the previously reported HCC cases [4].

## Main text

### Study cohort

The eligible cases included 225 Japanese patients (168 males and 57 females) with HCC who had underwent partial hepatectomy in the University of Miyazaki Hospital from February 1999 to October 2012. The patients included in this study were the same as in our previous report [4], as were the clinicopathological data (Additional file 1). Clinical parameters included age, gender, recurrence, tumor size, tumor multiplicity, infection with hepatitis B virus (HBV) and hepatitis C virus (HCV), serum alpha-fetoprotein (AFP) level, serum protein induced by vitamin K absence or antagonist II (PIVKA-II) level, Child–Pugh score, pre-operative therapy, post-operative therapy, TNM stage, and overall survival. Histological parameters included tumor differentiation, vascular invasion, capsular invasion, and cirrhosis. Tumor differentiation was assessed according to the World Health Organization classification.

### Immunohistochemistry

Serial sections, which were prepared from formalin-fixed, paraffin-embedded HCC blocks of 225 cases, were used for hematoxylin and eosin staining and MCT4 and GPC3 immunohistochemistry [4]. As the central portion of HCCs occasionally shows necrosis, a peripheral portion of HCC specimens associated with non-neoplastic liver tissue was randomly selected in each case. The sections were immunostained with anti-MCT4 rabbit polyclonal antibody (clone H-90; 1:200; Santa Cruz Biotechnology, Santa Cruz, CA, USA) or anti-GPC3 monoclonal antibody (GC33; 1 µg/ml) [18] as the primary antibody using the Leica Bond-Max III automated immunostainer (Leica Biosystems, Tokyo, Japan) according to the manufacturer’s instructions. Heat treatment for antigen retrieval was performed for 30 min prior to MCT4 and GPC3 immunohistochemistry. The primary antibody was omitted for negative controls in immunohistochemical analysis.

As reported in our previous studies [2, 4], we designated MCT4-positive HCC (MCT4+ HCC) and GPC3-positive HCC (GPC3+ HCC) as HCC cells with readily recognizable membranous MCT4 expression and HCC cells with readily recognizable membranous (circumferential, canalicular, and luminal) and/or cytoplasmic GPC3 expression, respectively. The evaluation of the immunohistochemical staining was performed by two or three independent researchers in a blinded fashion (A. O., K. Y., and/or H. K.).

### Statistical analysis

Fisher’s exact test or the Chi-square test was used for assessment of the relationship between variables. Statistical significance was assumed if *p* < 0.05. Data were analyzed by StatView 5.0 (SAS Institute Inc., Cary, NC, USA).

## Results

**Fig. 1:**
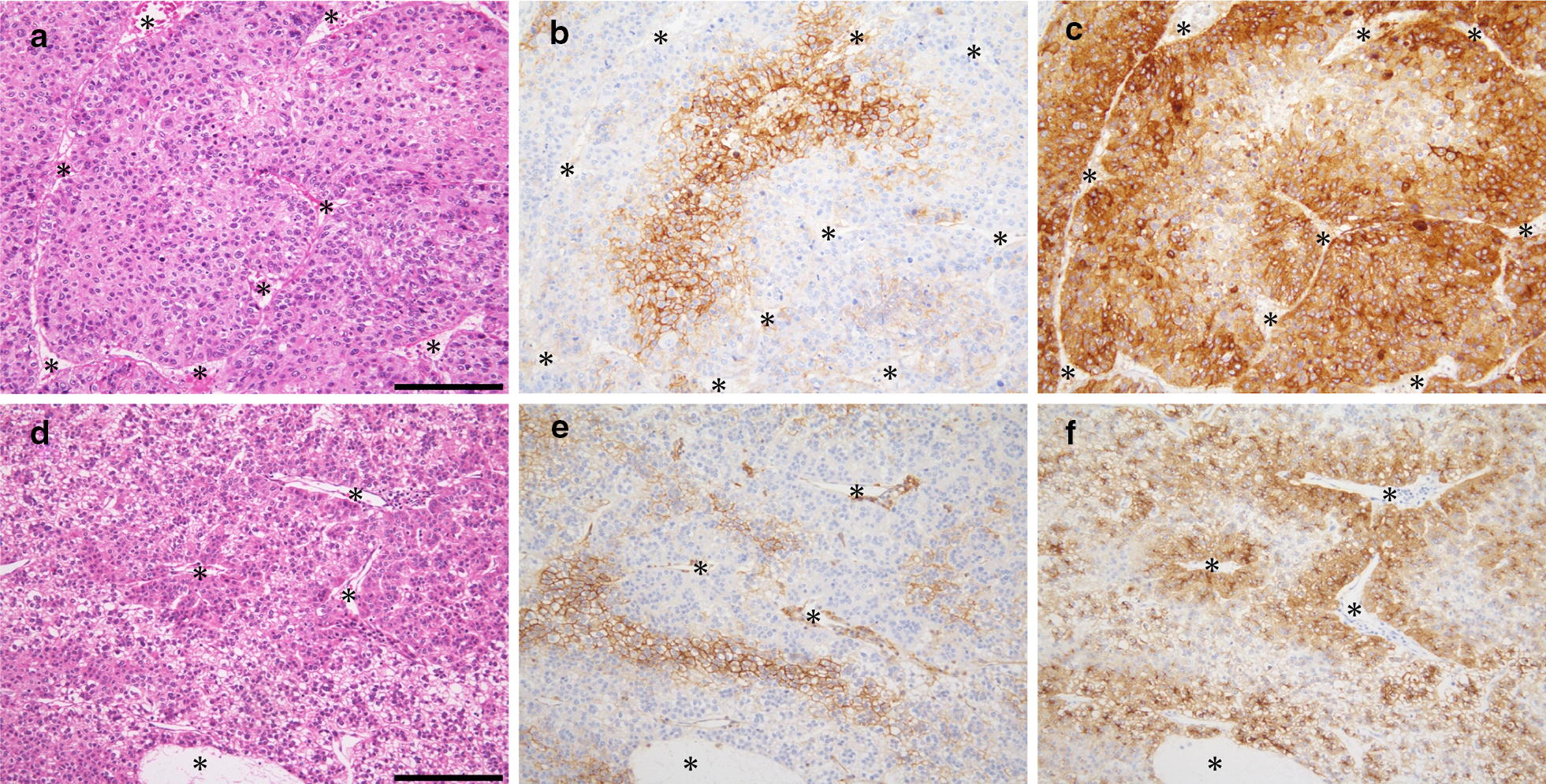
Representative cases of reciprocal interaction of MCT4 and GPC3 in HCC. Serial sections of two independent HCC cases (**a**–**f**) were stained with hematoxylin and eosin (**a**, **d**), anti-MCT4 antibody (**b**, **e**), and anti-GPC3 antibody (**c**, **f**). MCT4-positive HCC cells tended to be located distantly from vascular networks (asterisks), whereas GPC3-positive HCC cells tended to be present in the perivascular areas. Asterisks in **a**–**f** represent vascular networks. Scale bars, 100 µm

**Fig. 2:**
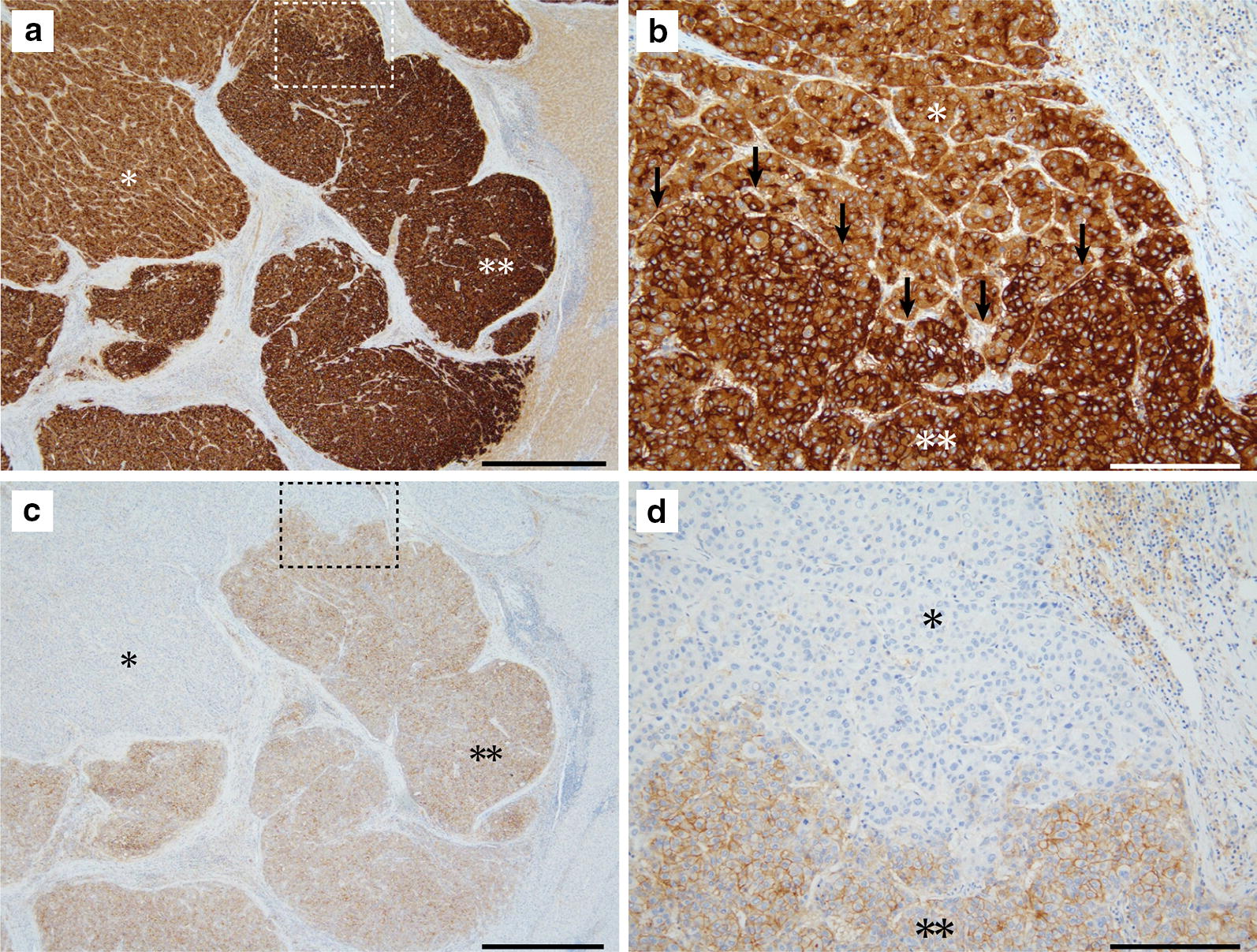
A representative case of synergistic interaction of MCT4 and GPC3 in HCC. Photos of GPC3-immunostained section are shown in the upper panel (**a** and **b**), and MCT4 immunostaining photos of the serial section are shown in the lower panel (**c** and **d**). **b** and **d** are magnified images of the dashed square in **a** and **c**, respectively. HCC cells were diffusely positive for GPC3 and partly positive for MCT4. The area with stronger GPC3 immunoreactivity is selectively positive for MCT4 (double asterisks) and GPC3-positive portion with weaker immunoreactivity is negative for MCT4 (asterisk). Arrows in **b** indicate the boundary of the asterisk-marked area and double asterisk-marked area. Scale bars are 1 mm (**a** and **c**) and 100 µm (**b** and **d**)

MCT4+ HCC and GPC3+ HCC were immunohistochemically identified in 21% (47 cases) and 84% (190 cases) of the 225 cases, respectively. The mean positive area of MCT4 and GPC3 was 20% (range of 1 to 80%; standard deviation, 26%) and 72% (range of 5 to 100%; standard deviation, 31%), respectively. MCT4+/GPC3+, MCT4+/GPC3-negative (GPC3−), MCT4-negative (MCT4−)/GPC3+, and MCT4−/GPC3− HCCs were observed in 44, 3, 146, and 32 cases, respectively (Additional file 1). Most MCT4+ HCC cases showed high GPC3 expression (94%, 44/47) as reported previously [4]. Of the 44 MCT4+/GPC3+ HCC cases, we observed reciprocal, synergistic, reciprocal and synergistic, or irrelevant expression pattern of MCT4 and GPC3, which represented 66% (29 cases), 11% (5), 2% (1), and 21% (9) of the cases, respectively (Additional file 2). Therefore, an intratumoral reciprocal (inverse) expression of MCT4 and GPC3 was the most frequent pattern in MCT4+/GPC3+ HCCs, in which HCC cells with increased MCT4 showed decreased GPC3 immunoreactivity and vice versa (Fig. 1). The central portions (areas distant from tumor vessels) of the tumor cell nests tended to show increased MCT4 and decreased GPC3 immunoreactivities, whereas the peripheral portions (areas adjacent to the tumor vessels) of the tumor nests tended to show decreased MCT4 and increased GPC3 immunoreactivities (Fig. 1). Among 30 HCC cases having intratumoral reciprocal expression pattern of MCT4 and GPC3 (29 reciprocal cases and 1 mixed reciprocal and synergistic case), 22 showed the reciprocal pattern in > 50% of the MCT4-positive area. The immunolocalization pattern of GPC3 in HCC showing the reciprocal interaction with MCT4 was circumferential membranous (5 cases), circumferential membranous and cytoplasmic (8), circumferential/canalicular membranous and cytoplasmic (5), canalicular membranous and cytoplasmic (1), or cytoplasmic (10). On the other hand, the synergistic interaction of MCT4 and GPC3 was suggested by increased MCT4 immunoreactivity in HCC that showed increased GPC3 immunoreactivity, and this pattern comprised 11% of the cases (Fig. 2). The immunolocalization pattern of GPC3 in HCC cells showing synergistic interaction with MCT4 was circumferential membranous pattern (1 case), circumferential membranous and cytoplasmic pattern (3), or circumferential/canalicular membranous and cytoplasmic pattern (1).

Using the MCT4+/GPC3+ HCC cases, except for one case showing mixed reciprocal and synergistic interactions of MCT4 and GPC3 (total 43 cases), we statistically analyzed the correlation between clinicopathological variables of the reciprocal HCC cases (29 cases) and those of the non-reciprocal ones (synergistic cases + irrelevant cases; 14 cases in total) (Table 1). Post-operative therapy was not included in the analysis as it was postulated to be unrelated to the GPC3/MCT4 expression pattern in the resected HCC. The reciprocal HCC cases were related to the existence of cirrhosis or absence of neoadjuvant therapy compared to the non-reciprocal HCC cases.

**Table 1:** Clinicopathological characteristics of patients with HCC according to the expression of MCT4 and GPC3

Parameters	Reciprocal n = 29	Non-reciprocal n = 14 [synergistic/irrelevant]	*p* value
Age			
< 60	12	7 [3/4]	0.5943
≥ 60	17	7 [2/5]	
Gender			
Male	18	13 [5/8]	0.0667
Female	11	1 [0/1]	
Recurrence			
New	25	10 [3/7]	0.4038
Recurrent	4	4 [2/2]	
Tumor size			
< 5 cm	15	7 [2/5]	0.9156
≥ 5 cm	14	7 [3/4]	
Tumor multiplicity			
Single	21	11 [4/7]	> 0.9999
Multiple	8	3 [1/2]	
HBV			
Positive	16	6 [3/3]	0.4490
Negative	13	8 [2/6]	
HCV			
Positive	6	5 [2/3]	0.2900
Negative	23	9 [3/6]	
No HBV or HCV			
Yes	8	3 [0/3]	> 0.9999
No	21	11 [5/6]	
Serum AFP			
High ≥ 14	25	10 [5/5]	0.4038
Low < 14	4	4 [0/4]	
Serum PIVKA-II^a^			
High ≥ 40	20	9 [4/5]	0.6369
Low < 40	8	5 [1/4]	
Child–Pugh score			
A	26	11 [5/6]	0.3728
B	3	3 [0/3]	
Pre-operative therapy			
Yes	3	5 [2/3]	*0.0452*
No	26	9 [3/6]	
Cirrhosis			
Yes	17	3 [1/2]	*0.0275*
No	12	11 [4/7]	
Capsular invasion			
Yes	21	9 [2/7]	0.5866
No	8	5 [3/2]	
Vascular invasion			
Yes	21	9 [4/5]	0.5866
No	8	5 [1/4]	
Tumor differentiation^b^			
Well	3	1 [0/1]	0.4239
Moderate	17	11 [4/7]	
Poor	9	2 [1/1]	
TNM stage^b^			
I	1	2 [0/2]	> 0.9999
II	7	1 [1/0]	
III	12	8 [1/7]	
IV	9	3 [3/0]	

Italic values were statistically significant

*HBV* hepatitis B virus, *HCV* hepatitis C virus, *AFP* alpha-fetoprotein, *PIVKA-II* protein induced by vitamin K absence or antagonist II

^a^n = 28

^b^Fisher’s exact test was performed for well—(TNM stage I + II) vs. moderately/poorly differentiated tumors (TNM stage III + IV). Numbers in brackets are synergistic/irrelevant HCC cases

## Discussion

We immunohistochemically demonstrated that most (94%) of MCT4+ HCC cases in our cohort showed GPC3 positivity, and nearly 80% of MCT4+ HCC cases exhibited reciprocal or synergistic expression pattern between MCT4 and GPC3. Thus, the expression of MCT4 in HCC cells might be influenced by GPC3 expression and vice versa. Of note, 68% of MCT4+/GPC3+ HCC cases demonstrated reciprocal interaction of both markers. These findings may provide a novel therapeutic approach for MCT4+ HCC; dual targeting of MCT4 and GPC3 may achieve a better antitumor effect for MCT4+ HCC.

In this study, we used the custom-made anti-GPC3 antibody GC33, which is a mouse monoclonal antibody that recognizes human GPC3. Humanized GC33 (codrituzumab) may serve as a treatment option for HCC because it has a significant antitumor activity to HCC cells in vivo via antibody-dependent cellular cytotoxicity [19, 20]. We anticipate the essentially same results as this study if a commercially available anti-GPC3 antibody (1G12) was used for the immunostaining, as the immunolocalization pattern of GPC3 detected by 1G12 is completely the same as that by GC33 [2].

The mechanism of reciprocal interaction of MCT4 and GPC3 in HCCs remains unknown. In the tumor areas showing reciprocal interaction of MCT4 and GPC3, MCT4 was likely induced by the hypoxic tumor microenvironment because MCT4+ HCC cells were observed primarily in the central portions of tumor nests distant from the tumor vessels. In fact, we previously showed that MCT4+ HCC cells were present near necrotic portions, and those tumor cells tended to be positive for the hypoxia marker carbonic anhydrase IX [4]. This finding is likely reasonable, considering that MCT4 can be induced by hypoxia. On the other hand, the mechanism underlying the expression of GPC3 in HCCs is not well understood; however, considering the reciprocal interaction of MCT4 and GPC3, GPC3 expression might also be regulated by a hypoxic tumor microenvironment, which could decrease GPC3 expression in HCC cells. The expression of *GPC3* is silenced partly by promoter hypermethylation in some cancers [21, 22], and DNA hypermethylation can be induced by tumor hypoxia [23]. Alternatively, *GPC3* transcription in HCC may be suppressed by transcription factor zinc fingers and homeoboxes 2 (ZHX2), a well-known repressor of the *GPC3* gene [24, 25], in a hypoxic condition.

Although the reciprocal pattern was predominant, 11% of the cases showed a synergistic expression pattern of MCT4 and GPC3. The mechanism underlying the synergistic interaction of MCT4 and GPC3 in HCC also remains unclear. In the areas of tumors showing synergistic interaction of MCT4 and GPC3, concomitant cell surface immunoreactivities of MCT4 and GPC3 were observed as reported previously [4]. This finding suggested the interaction between MCT4 and GPC3 on the HCC cell surface. Evidence indicates that GPC3 co-localizes with GLUT4, a glucose transporter [26], suggesting that GPC3 may facilitate glucose uptake through GLUT4. Thus, in a subset of HCC cases, GPC3 may interact with MCT4 and GLUT4 on the cell surface and facilitate their functions, allowing HCC cells to easily adapt to hypoxic microenvironments and accelerate the invasive phenotype with CD147, an inducer of matrix metalloproteases frequently co-existing with MCT4 [13–15].

Based on statistical analysis, the reciprocal interaction of MCT4 and GPC3 tended to be observed in non-treated HCCs derived from cirrhosis. Thus, severe cell damage induced by adjuvant therapy in HCC may disturb the reciprocal relationship and may result in a synergistic or irrelevant interaction of MCT4 and GPC3; however, this hypothesis remains highly speculative.

In conclusion, we immunohistochemically explored the intratumoral relationship between MCT4 and GPC3 expression in HCC. Although the mechanism underlying the interaction of these molecules in HCC is currently unknown, the observed phenomena may have implications in the development of therapeutic strategies targeting MCT4 and GPC3 in HCC.

## Limitations

- Expression of MCT4 and GPC3 in HCC cells was immunohistochemically evaluated using formalin-fixed, paraffin-embedded HCC tissue blocks from 225 cases. In each case, one tumor block was randomly selected from the maximal section of the tumor. The expression status of MCT4 and GPC3 was not evaluated in whole tumor sections.
- Prognostic differences between patients with “reciprocal” and “non-reciprocal” HCC could not be statistically evaluated owing to the small sample size.
- With respect to the expression regulation mechanism of MCT4 and GPC3 in HCC cells, we could not sufficiently explain the mechanism with this morphological study.

## Supplementary information

**Additional file 1.** Clinicopathological data of patients with HCC.

**Additional file 2.** Intratumoral expression patterns of MCT4 and GPC3 in 44 cases of MCT4+ GPC3+ HCC.

## Data Availability

The datasets used and/or analyzed during the current study are available from the corresponding author on request.

## Abbreviations

MCT4: monocarboxylate transporter 4
GPC3: glypican-3
HCC: hepatocellular carcinoma
HBV: hepatitis B virus
HCV: hepatitis C virus
AFP: alpha-fetoprotein
PIVKA: protein induced by vitamin K absence or antagonist II
ZHX2: zinc fingers and homeoboxes 2
