# Supplementary material for: Intratumoral reciprocal expression of monocarboxylate transporter 4 and glypican-3 in hepatocellular carcinomas
Source: BMC Res Notes. 2019 Nov 9;12:741. doi: 10.1186/s13104-019-4778-y (PMC6842510; doi:10.1186/s13104-019-4778-y)
Supplement: Supplementary file 1 — Additional file 1. Clinicopathological data of patients with HCC. [file 13104_2019_4778_MOESM1_ESM.docx]

Additional file 1. Clinicopathological data of patients with HCC

Variable n Mean ± SD/Median (Range)

Age (years) 225 65.5 ± 11.0/68.0 (18–86)

Gender, Male/Female 168/57

HBV/HCV/Both/None 68/90/4/71

Newly onset/Recurrence 183/42

Tumor size (cm) 225 4.8 ± 3.4/4.0 (0.8–19)

Tumor number, single/multiple 175/50

Serum AFP (ng/ml) 225 12614 ± 89409/28 (1–1121170)

Serum PIVKA-II (mAU/ml) 223 7173 ± 34215/124 (7–443000)

Child-Pugh score, A/B 196/29

Pre-operative therapy 55

Post-operative therapy 40

Tumor differentiation

Well/Moderate/Poor 82/123/20

Vascular invasion, +/– 118/107

Capsular invasion, +/– 140/85

Cirrhosis, +/– 109/116

TNM Stage, I/II/III/IV 23/80/81/41

MCT4 positive/GPC3 positive 44

MCT4 positive/GPC3 negative 3

MCT4 negative/GPC3 positive 146

MCT4 negative/GPC3 negative 32

Abbreviations: HCC, hepatocellular carcinoma; SD, standard deviation; HBV, hepatitis B virus; HCV, hepatitis C virus; AFP, alpha-fetoprotein; PIVKA-II, protein induced by vitamin K absence or antagonist II; AU, Anson unit; +/–, present/absent; MCT4, monocarboxylate transporter 4; GPC3, glypican-3
