# Supplementary material for: Intratumoral reciprocal expression of monocarboxylate transporter 4 and glypican-3 in hepatocellular carcinomas
Source: BMC Res Notes. 2019 Nov 9;12:741. doi: 10.1186/s13104-019-4778-y (PMC6842510; doi:10.1186/s13104-019-4778-y)
Supplement: Supplementary file 2 — Additional file 2. Intratumoral expression patterns of MCT4 and GPC3 in 44 cases of MCT4+ GPC3+ HCC. [file 13104_2019_4778_MOESM2_ESM.docx]

Additional file 2. Intratumoral expression patterns of MCT4 and GPC3 in 44 cases of MCT4+GPC3+ HCC

Relative expression of MCT4 and GPC3 n (%)

Reciprocal 29 (66%)

Synergistic 5 (11%)

Reciprocal and synergistic 1 (2%)

Irrelevant 9 (21%)
